# Aldehyde dehydrogenase 2 Glu504Lys variant predicts a worse prognosis of acute coronary syndrome patients

**DOI:** 10.1111/jcmm.13536

**Published:** 2018-02-14

**Authors:** Chang Pan, Yu Zhao, Yuan Bian, Rui Shang, Jia‐li Wang, Li Xue, Shu‐jian Wei, He Zhang, Yu‐guo Chen, Feng Xu

**Affiliations:** ^1^ Department of Emergency Medicine and Chest Pain Center Qilu Hospital Shandong University Jinan China; ^2^ Key Laboratory of Cardiovascular Remodeling and Function Research Ministry of Education and Ministry of Public Health of People's Republic of China Qilu Hospital Shandong University Jinan China; ^3^ Institute of Emergency and Critical Care Medicine Shandong University Jinan China; ^4^ Key Laboratory of Emergency and Critical Care Medicine of Shandong Province Qilu Hospital Shandong University Jinan China

**Keywords:** acute coronary syndrome, aldehyde dehydrogenase 2, Glu504Lys variant, prognosis

## Abstract

Aldehyde dehydrogenase 2 (ALDH2) Glu504Lys variant was an independent risk factor for acute coronary syndrome (ACS). However, there are lacking researches about the relationship between the variant and prognosis of ACS. In the prospective study, 377 ACS patients were grouped into the wild‐type (*1/*1) and the mutation (*2/*2 + *1/*2) groups according to genotype detection. Compared with the wild‐type group, incidences of major adverse cardiac events (MACE) and cardiac death were both higher in the mutation group (9.2% vs 21.0%, *P *=* *.002; 5.2% vs 12.2%, *P *=* *.026); the MACE‐free and the cardiac‐death‐free cumulative survival rates were obviously lower in the mutation group. Moreover, the mutant genotypes were associated with significantly increased risk of MACE and cardiac death (HR 2.443, 95%CI: 1.390‐4.296, *P *=* *.002; HR 2.727, 95%CI: 1.303‐5.708, *P *=* *.008). These results suggested that ALDH2 Glu504Lys variant could predict a worse prognosis of ACS patients.

## 1 INTRODUCTION

Acute coronary syndrome (ACS) is the most common cause of death, accounting for 40% of all deaths worldwide. The occurrence of ACS is the result of a complex combination of genetic and environmental factors.

As the pivotal enzyme of traditional alcohol metabolism, aldehyde dehydrogenase 2 (ALDH2) has a significant functional single‐nucleotide polymorphism, the rs671‐Glu504Lys variant, showing dramatically reduced activity compared with the wild‐type.^1^ The rs671 variant exists in 30%‐50% of East Asians (Chinese, Japanese and Korean) and 6% of the world population. ALDH2 with different enzymatic activities exerts different effects on inflammation and oxidative stress. ALDH2 with high activity plays a cardinal role in ameliorating oxidative stress and oxidizing endogenous aldehyde products, such as malondialdehyde, 4‐hydroxy‐2‐nonenal and environmental aldehydes like acrolein.^2^

Previous studies conducted in China, Japan and Korea demonstrated that ALDH2 Glu504Lys variant was closely related to ACS or myocardial infarction (MI) and that the mutant genotypes (*2/*2 and *1/*2) were independent risk factors for ACS in East Asians.^2^, ^3^, ^4^ Moreover, a genome‐wide association study of common SNPs confirmed that *aldh2* is the susceptibility gene of coronary artery disease (CAD).^5^ However, to date, there are still lacking researches about the relationship between the Glu504Lys variant and prognosis of ACS.

In our prospective cohort study, we investigated the prognostic role of ALDH2 Glu504Lys variant in ACS patients.

## 2 MATERIALS AND METHODS

### 2.1 Study protocol

The prospective study was approved by the Ethics Committee of Qilu Hospital of Shandong University, and all participants were enrolled after written informed consent. The study consecutively enrolled 377 hospitalized ACS patients who underwent coronary angiography from January 2007 to July 2009 in Qilu Hospital of Shandong University. All the patients enrolled were Han Chinese from Shandong Province of China. The inclusion criteria were as follows: (i) clinical manifestations, electrocardiograph (ECG) alteration and changes of myocardial enzymology met the diagnosis criteria for unstable angina pectoris, non‐ST‐segment elevation myocardial infarction (NSTEMI) and ST‐segment elevation myocardial infarction (STEMI) formulated by American College of Cardiology/American Heart Association; (ii) the coronary angiography exhibited at least one coronary artery stenosis ≥50%; (iii) there were no hereditary relationships among patients. The patients were excluded if they suffered from malignancy. The blood samples of patients were collected within 24 hours after admission.

### 2.2 Basic clinical data

Detailed in Appendix S1.

### 2.3 Genotype detection and group

Detailed in Appendix S1.

### 2.4 Follow‐up

We followed up patients meeting study eligibility criteria by means of telephone contacts and recorded their clinical events. The major adverse cardiac events (MACE), including cardiac death, MI, revascularization and new heart failure, were regarded as the primary end‐point. The researchers who adjudicated the clinical end‐points of the patients were blinded to the genotype assessments.

### 2.5 Statistical analyses

All statistical analyses were performed with SPSS 17.0 (SPSS Inc., Chicago, IL, USA). Numerical variables were analysed by Student's *t* test or Mann‐Whitney *U* test; categorical variables were analysed by Chi‐square test or Fisher's exact test. Hardy‐Weinberg equilibrium was used to assess the representativeness of the patients. Cumulative mortality was estimated by Kaplan‐Meier analysis, and the patients lost during follow‐up were censored in the analysis. Numerical variables in non‐normally distributed variables were enrolled in survival analysis after logarithm transformation. To estimate the independent prognostic utility of ALDH2 mutant genotypes on MACE and cardiac death, a Cox proportional hazard model was used to calculate the hazard ratios (HR) and the 95% confidence intervals (CI). A series of data were enrolled in Cox regression analysis, including ALDH2 genotype, age, gender, body mass index (BMI), levels of plasma lipids, smoking history, family history of CAD, hypertension, diabetes, prior MI, prior revascularization, admission diagnosis, number of diseased coronary arteries, revascularization and number of coronary arteries treated with percutaneous coronary intervention (PCI). *P *<* *.05 was considered statistically significant.

## 3 RESULTS

### 3.1 Demographic characteristics of ACS patients

According to ALDH2 genotyping, the number of homozygotes with *1/*1 and *2/*2 and heterozygotes with *1/*2 was 229 (60.7%), 12 (3.2%) and 136 (36.1%), respectively. The polymorphisms did not deviate from a Hardy‐Weinberg equilibrium (*P *=* *.125). And, moreover, the 377 ACS patients were divided into the wild‐type (*1/*1, n* *=* *229) and the mutation groups (*2/*2 + *1/*2, n* *=* *148). Between the 2 groups, there was no statistically significant difference in the demographic characteristics, including age, gender, BMI, blood lipids levels, smoking, hypertension, diabetes, family history of CAD, prior MI, prior revascularization, and diagnosis on admission (Table S1). Yet, compared with the mutation group, the proportion of patients who drank at least 1 day a week was higher (31.8% vs 44.1%, *P *=* *.017) with more average daily alcohol consumption in the wild‐type group (Table S1).

### 3.2 Angiography and revascularization of ACS patients with different ALDH2 genotypes

Between the 2 groups, there was no significant difference in the number of lesion coronary arteries. However, in the mutation group, the incidence of left main (LM) coronary artery disease was higher than that in the wild‐type group (18.2% vs 10%, *P *=* *.033). Yet, other angiography findings, including the number of left anterior descending branch (LAD), left circumflex branch (LCX) and right coronary artery (RCA) with lesions, revascularization, the number of revascularization vessel >1 and TIMI3 grade after PCI, did not differ by ALDH2 genotypes (*P *>* *.05) (Table 1).

**Table 1:** Angiography and revascularization of ACS patients with different ALDH2 genotypes

	*1/*1 (n* *=* *229)	*1/*2 + *2/*2 (n* *=* *148)	*P*‐value
Lesion vessel number			NS
1 (n, %)	55 (24%)	39 (26.4%)	
2 (n, %)	69 (30.1%)	43 (29.1%)	
3 (n, %)	105 (45.9%)	66 (44.6%)	
Lesion vessel			
LM (n, %)	23 (10%)	27 (18.2%)	.033
LAD (n, %)	184 (80.3%)	124 (83.8%)	NS
LCX (n, %)	146 (63.8%)	90 (60.8%)	NS
RCA (n, %)	167 (72.9%)	98 (66.2%)	NS
Revascularization (n, %)	187 (81.7%)	130 (87.8%)	NS
Revascularization vessel >1 (n, %)	112 (48.9%)	81 (54.7%)	NS
TIMI3 grade after PCI (n, %)	219 (95.6%)	139 (93.9%)	NS

Data are shown as frequency number or %.

ACS, acute coronary disease; LM, left main; LAD, left anterior descending; LCX, left circumflex; RCA, right coronary artery; PCI, percutaneous coronary intervention; ALDH2, aldehyde dehydrogenase 2.

### 3.3 Follow‐up

The follow‐up was accomplished in 95.2% of the patients with the median follow‐up time as 38 months, ranging from 24 to 55 months. The medications, including aspirin, clopidogrel, statins, β‐blockers and ACEI/ARB, were similar between the wild‐type and the mutation groups. At the end of follow‐up, the overall incidence of MACE was 13.8% and the all‐cause mortality was 9%, of which the incidence of cardiac death was 8%. In the mutation group, the incidences of MACE and cardiac death were both higher than those in the wild‐type group (21.0% vs 9.2%, *P *=* *.002; 12.2% vs 5.2%, *P *=* *.026).

### 3.4 Survival analysis

**Figure 1:**
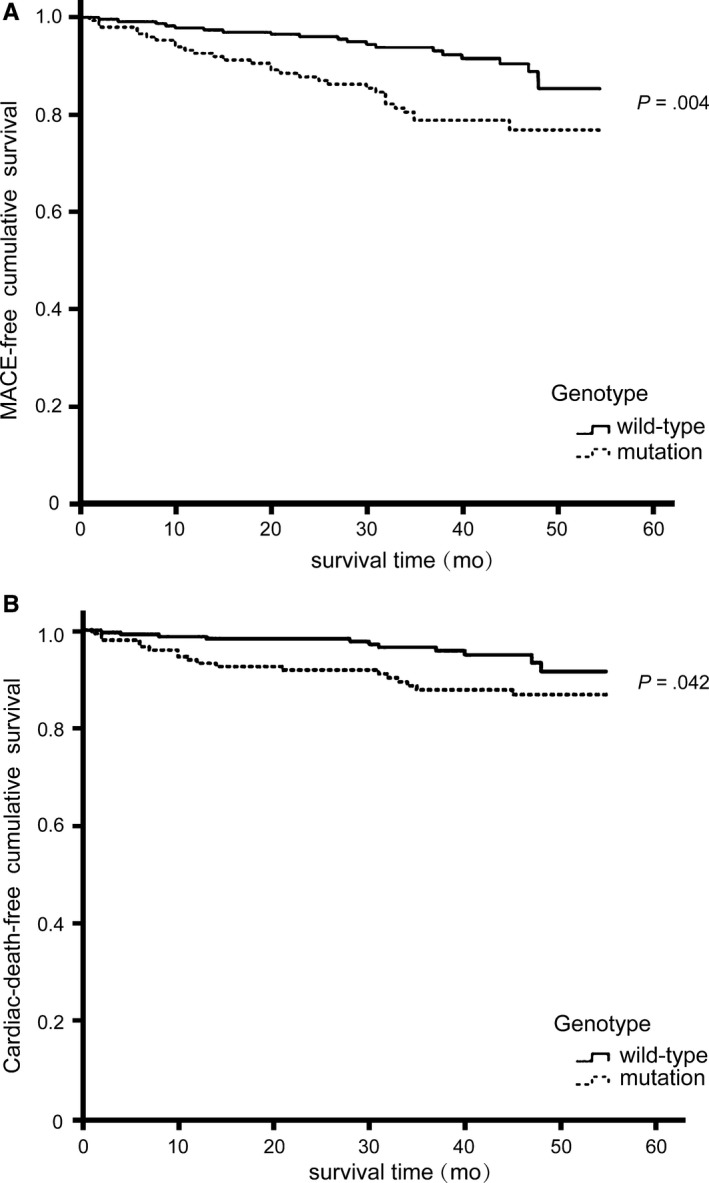
Kaplan‐Meier analysis showed that MACE‐free cumulative survival rates (A) and cardiac‐death‐free cumulative survival rates (B) in patients with ALDH2 mutant genotypes were obviously lower than those in patients with wild genotype (*P *<* *.05). MACE, major adverse cardiac events; ALDH2, aldehyde dehydrogenase 2

Kaplan‐Meier analysis revealed that the MACE‐free and cardiac‐death‐free cumulative survival rates in the wild‐type group were obviously higher than those in the mutation group (*P *<* *.05) (Figure 1). Furthermore, Cox regression analysis confirmed that the ALDH2 mutant genotype was an independent predictive factor of MACE and cardiac death in ACS patients (HR 2.443, 95%CI: 1.390‐4.296, *P *=* *.002; HR 2.727, 95%CI: 1.303‐5.708, *P *=* *.008).

## 4 DISCUSSION

Coronary artery disease or ACS is an important public health issue and the most common cause of death, as well as a severe financial burden worldwide. Discovery of genetic risk factors will help improve the progression of ACS and help find new effective drug targets. Previous studies have demonstrated the relationship between ALDH2 Glu504Lys variant and the risk of CAD or ACS.^2^, ^3^, ^4^, ^5^ However, whether the variant was associated with the prognosis of ACS patients had never been investigated. In the single‐centre prospective cohort study, we for the first time investigated the correlation between ALDH2 Glu504Lys variant and the prognosis of ACS patients. We demonstrated that ACS patients with the mutant ALDH2 (*2/*2 and *1/*2) had an increased risk of MACE and cardiac death; ALDH2 Glu504Lys variant could predict a worse prognosis of ACS patients.

As the most efficient enzyme for metabolizing ethanol‐derived aldehydes, ALDH2 also participates in metabolizing other short‐chain aliphatic aldehydes, some polycyclic aldehydes and aromatic aldehydes.^2^ Heterozygotes with ALDH2 *1/*2 have dramatically lower than 50% and homozygotic individuals with ALDH2 *2/*2 have lower than 1%‐4% of the homozygotes with ALDH2 *1/*1.^2^ Therefore, individuals with ALDH2 *2 allele exhibit slower aldehyde metabolism after alcohol consumption and they may reduce their alcohol intake.

As to the underlying mechanisms of how the variant influenced the prognosis of ACS, it might be related to the effect of ALDH2 on detoxification of toxic aldehydes, inhibition of oxidative stress and inflammation, and so on.^6^, ^7^, ^8^, ^9^, ^10^, ^11^, ^12^, ^13^, ^14^, ^15^ In animal models of MI, enhancement of ALDH2 activity by Alda‐1 or overexpression of ALDH2 could significantly alleviate ischaemia/reperfusion injury, reduce infarct size of heart, and improve ventricular function and heart failure outcome after MI or in post‐MI cardiomyopathy, via reducing the toxic effects of aldehydic overload, inhibiting reactive oxygen species generation and regulating autophagy.^8^, ^9^, ^10^, ^11^, ^12^, ^13^ Moreover, ALDH2 performed an important function on alleviating cardiomyocytes apoptosis and myocardial senescence through protecting mitochondrial function and correction of autophagy.^14^, ^15^

However, there were some limitations in our study. The sample size of our study was relatively small, and we have not yet replicated our findings in another independent sample. To further validate the association between ALDH2 Glu504Lys variant and the prognosis of ACS patients, and to reveal the underlying mechanism, a multi‐centre long‐term prospective study with more patients should be performed in the future.

In the prospective cohort study, we investigated the prognostic role of ALDH2 Glu504Lys variant in ACS patients and demonstrated a significant association between the variant and an increased risk of MACE and cardiac death of ACS patients. We hope our findings could help to stratify the high‐risk ACS patients and guide the precision treatment.

## CONFLICTS OF INTEREST

The authors confirm that there is no conflict of interests.
