# Supplementary material for: Aldehyde dehydrogenase 2 Glu504Lys variant predicts a worse prognosis of acute coronary syndrome patients
Source: J Cell Mol Med. 2018 Feb 14;22(4):2518–22. doi: 10.1111/jcmm.13536 (PMC5867093; doi:10.1111/jcmm.13536)
Supplement: Supplementary file 1 [file JCMM-22-2518-s001.docx]

**Supplementary Materials**

**THIS FILE INCLUDES:**

**Supplementary-Table S1**: Demographic characteristic of all ACS patients with different ALDH2 genotypes.

**Supplementary Materials and Methods**

**Table S1 Demographic characteristic of all ACS patients with different ALDH2 genotypes**

|  | *1/*1  (*n* = 229) | *1/*2+*2/*2  (*n* = 148) | *P*-value |
| --- | --- | --- | --- |
| Age (years)  Male gender (*n*, %)  BMI (kg/m^2^)  TC (mmol/L)  TG (mmol/L)  LDL-C (mmol/L)  HDL-C (mmol/L)  Smoking (*n*, %)  Alcohol consumption  ≥ 1 day per week  Alcohol consumption (g/day)  Hypertension (*n*, %)  Diabetes mellitus (*n*, %)  Family history of CAD  Prior MI (*n*, %)  Prior revascularization (*n*, %)  Diagnosis on admission  NSTEMI (*n*, %)  STEMI (*n*, %) | 61.9 ± 10.8  167 (72.9%)  25.3 ± 3.4  4.77 ± 1.04  1.66 (1.08%)  2.92 ± 0.92  1.26 ± 0.51  123 (53.7%)  101 (44.1%)  2.0 (39.76)  124 (54.2%)  54 (23.6%)  30 (13.1%)  37 (16.2%)  11 (4.8%)  24 (10.5%)  44 (19.2%) | 61.2 ± 10.5  115 (77.7%)  25.5 ± 3.3  4.62 ± 1.13  1.61 (1.05%)  2.78 ± 0.95  1.28 ± 0.59  82 (55.4%)  47 (31.8%)  1.0 (6.86)  83 (56.1%)  38 (25.7%)  22 (14.9%)  14 (9.5%)  8 (5.4%)  8 (5.4%)  31 (20.9%) | NS  NS  NS  NS  NS  NS  NS  NS  0.017  < 0.001  NS  NS  NS  NS  NS  NS  NS |

Numerical variable data are shown as mean ± SD, median or interquartile range; categorical variable data are presented as frequency number or %.

ACS: acute coronary disease; BMI: body mass index; TC: total cholesterol; TG, triglycerides; LDL-C: low-density lipoprotein cholesterol; HDL-C: high-density lipoprotein cholesterol; CAD: coronary artery disease; NSTEMI: non-ST segment elevated myocardial infarction; STEMI: ST segment elevated myocardial infarction

**Supplementary Materials and Methods**

**Basic clinical data**

We recorded demographic data of all subjects by case report forms, including age, gender, the body mass index (BMI), smoking, alcohol consumption, family history of CAD, diabetes, hypertension, prior MI, prior revascularization, diagnosis on admission, angiography findings, medicine treatment, and levels of plasma lipids. Blood lipids included high-density lipoprotein cholesterol (HDL)-C, low-density lipoprotein cholesterol (LDL)-C, total cholesterol (TC) and triglyceride (TG). Concentrations of fasting plasma HDL-C, LDL-C, TC and TG were measured by the standard biochemical methods with an Olympus AU5400 Autoanalyzer (Dallas, Texas, USA).

Drinking frequency (drinking days per week) and the average daily alcohol consumption were recorded. 40 ml of liquor, 355 ml of beer or 118 ml of wine were considered to contain the same amount of alcohol (approximately 14 g).

**Genotype detection and group**

DNA was extracted from the peripheral venous blood, and the method of ALDH2 gene polymorphism detection was the same as previously described[1]. Briefly, genome DNA was extracted from 200μl of venous blood firstly using a commercial DNA isolation kit (TIANGEN, Peking, China), and then the genome DNA underwent polymerase chain reaction (PCR) with the primers as follows: 5′-GTCAACTGCTATGATGTGTTTGG-3′, 5′-CCACCAGCAGACCCTCAAG-3′. After purification, the PCR products were sequenced by Invitrogen Corporation (Shanghai, China). According to the results of genotype detection, these patients were grouped into the wild-type (*1/*1) and the mutation group (*2/*2+*1/*2).

**Reference**

1. **Xu F, YG Chen, L Xue, *et al***. Role of aldehyde dehydrogenase 2 Glu504lys polymorphism in acute coronary syndrome*.* *J Cell Mol Med*. 2011;15:1955-62.
